# Moderate sedation with single-dose remimazolam tosilate in elderly male patients undergoing transurethral resection of the prostate with spinal anesthesia: a prospective, single-arm, single-centre clinical trial

**DOI:** 10.1186/s12871-022-01788-1

**Published:** 2022-08-04

**Authors:** Tang-yuan-meng Zhao, Di Chen, Hu Sun, Zhi-xin Xu, Song Lyu, Tao Wang, Li-li Liu

**Affiliations:** The Second Affiliated Hospital of Hainan Medical University, Hai Kou, China

**Keywords:** Remimazolam, Spinal anesthesia, Elderly patients, TURP

## Abstract

**Background:**

Remimazolam tosilate (RT) is a newly listed benzodiazepine for sedation and anesthesia featuring quick onset of effects, short maintenance and recovery times, which is currently under research. This trial was conducted to determine the median effective dose (ED_50_) and the 95% effective dose (ED_95_) of single-dose remimazolam for moderate sedation in elderly patients undergoing transurethral resection of the prostate (TURP) under spinal anesthesia, and to evaluate its efficacy and safety.

**Methods:**

Thirty male patients aged 65–80 years old were recruited for selective TURP. Remimazolam was administered intravenously to pain-free patients (VAS score < 1) within 1 min of successful spinal anesthesia by the same anesthesiologist. We used modified Dixon’s up-and-down sequential allocation method to determine the ED_50_ and ED_95_ of the agent with an initial dosage of 0.1 mg/kg. Successful sedation was defined as an MOAA/S score ≤ 3 and above 1. A score of > 3 was deemed as failed sedation. Recruitment continued until ten independent pairs (from successful sedation to failed sedation) would give a reliable estimation of the ED_50_ and ED_95_ of RT and their 95% confidence intervals.

**Results:**

The ED_50_ of remimazolam was 0.063 (95% C.I. 0.045–0.073) mg/kg. Its ED_95_ was 0.079 (95% C.I. 0.07–0.137) mg/kg. Remimazolam was safe in its application.

**Conclusions:**

A single-dose of RT proves to be safe for assisted sedation during TURP in elderly male patients under spinal anesthesia with a lower incidence of adverse events. Its ED_50_ and ED_95_ were 0.063 mg/kg and 0.079 mg/kg, respectively.

**Trial registration:**

http://www.chictr.org.cn (ChiCTR2100051912).

**Supplementary Information:**

The online version contains supplementary material available at 10.1186/s12871-022-01788-1.

## Background

Globally, population aging is driven by declined fertility and improved longevity [1]. Due to cardiac and respiratory dysfunction, elderly patients are facing higher risks of surgery and anesthesia [2]. Anesthesiologists must consider the physiological features of the elderly to ensure perioperative safety. In the world, more and more elderly patients require surgical operation due to debilitating physical functions, a large proportion of which are with prostate hyperplasia [3]. Regional anesthesia is increasing in popularity. Intraspinal anesthesia has become the optimal anesthetic method for elderly patients undergoing transurethral resection of the prostate (TURP). Unlike general anesthesia, regional block at a lower spinal plane has little effect on cardiopulmonary functions. However, excessive sedation may cause adverse effects in the aged group [4, 5].

Remimazolam tosilate (RT) is a newly listed benzodiazepine for sedation and anesthesia characterized by quick onset of effects, short maintenance and recovery times, which is under ongoing research. Not accumulating in tissues, it metabolizes without affecting liver and kidney function or causing major side effects. Clinically, remimazolam has been safely applied in endoscopic procedures, and its sedative effects are easily reversed with flumazenil [6–8]. The initial positioning of remimazolam on the market was intraoperative sedation for non-tracheal intubation general anesthesia for endoscopic operations, and as drug research continued, the application of remimazolam for the increasing regional anesthetic modalities became more common. Moreover, the surgical position of our trial patient was a lithotomy position, which was more convenient for airway management during patient anesthesia compared to intraoperative sedation for gastroscopic surgery.

As a sedative drug for surgery anesthesia, there is no report on its application among the elderly undergoing TURP with spinal anesthesia. Our study aimed to determine the median effective dose (ED_50_) and the 95% effective dose (ED_95_) of single-dose remimazolam tosilate for moderate sedation during the target procedure, and to evaluate its efficacy and safety.

## Methods

### Ethics and registration

This study was approved by the Clinical Research Ethics Committee of the Second Affiliated Hospital of Hainan Medical University (reference number 2021–024-02, 20/5/2021) and registered at http://www.chictr.org.cn (ChiCTR2100051912, 9/10/2021). The study protocol was performed in the relevant guidelines. The trail was conducted in accordance with the principles of the Institutional Research Board of the authorized hospital. Written informed consent was obtained from all patients.

### Patient inclusion and exclusion criteria

At present, there are few reports on applying remimazolam in regional anesthesia. Considering the sexual needs of patients, the intraoperative use of tourniquets, and the impact of pain on hemodynamics, we designed a single-arm trial and limited the study subjects to elderly patients who planned to undergo TURP with spinal anesthesia. The study was carried out in the Second Affiliated Hospital of Hainan Medical College. Thirty patients who were scheduled to receive elective TURP were recruited. To ensure the test homogeneity, the inclusion criteria of the patients were age between 65 and 80 years old, American Society of Anesthesiologist (ASA) physical status I or II and a body mass index (BMI) between 19 and 30 kg/m^2^. Patients with history of alcoholism or allergy to local or general anesthetics, puncture wound infection, coagulation disorders, psychiatric or neurological diseases were excluded.

### Pretreatment and technique

We diluted 36 mg of remimazolam tosilate (developed by Jiangsu Hengrui Medicine Co. Ltd., China, 201031AK, YBH03052019) with 72 ml of 0.9% sodium chloride injection to a concentration of 0.5 mg/ml in schering bottles. We prepared 5 ml of 2% lidocaine (Hubei Tiansheng Pharmaceutical Co. Ltd.,China, H42021839) for local anesthesia at the puncture site and 3 ml of 0.5% ropivacaine (AstraZeneca AB®, H20140764, LBUD) for subarachnoid block. Combined spinal-epidural anesthesia puncture was performed with AS-E/SII needles (Jiangxi Hongda Medical Equipment Group Ltd., China; epidural anesthesia needle: 1.6 × 80 mm; subarachnoid anesthesia puncture needle: 0.5 × 113 mm, 20,200,812, 20,150,075). Notably, since isobaric solutions of ropivacaine were chosen for subarachnoid block, we diluted ropivacaine with patients’ own cerebrospinal fluid (CSF). The above-mentioned concentration of ropivacaine was a diluted one.

The night before the patient's surgery, the anesthesiologist will perform a pre-anesthesia visit. In addition to the usual physical examination, the anesthesiologist will carefully evaluate the patient's past and present medical history, laboratory tests, and imaging studies. This will determine whether the patient is suitable for our trial. In addition, based on the surgical characteristics of the Second Affiliated Hospital of Hainan Medical University, there are a significant number of elderly patients undergoing TURP to satisfy the screening and trial.

All patients fasted for at least 8 h before the surgery and were made sure by anesthesiologists that no unnecessary substance was given preoperatively, including benzodiazepines and alcohol. On arrival in the operating room, patients were connected to a monitor (the Bene View N15 OR monitor, Mindray Biomedical Electronics Co., Shenzhen, China) for continuous monitoring of electrocardiogram (ECG), noninvasive blood pressure (NIBP) including systolic blood pressure (SBP) and diastolic blood pressure (DBP), blood oxygen saturation (SpO_2_), respiratory rate (RR) and heart rate (HR). The monitoring was repeated three times, and the mean of each indicator was determined as the baseline value. Ideally, patients breathed in room air throughout the course without inhaling pure oxygen. For patient safety, we were prepared for artificial ventilation. When patients’ vital signs were stable, peripheral veins were punctured to insert indwelling catheter. Then, 300 ml of Ringer’s solution was administered intravenously.

In order to evaluate the effect of rimazolam on patients’ liver and kidney function, we collected laboratory tests to routinely evaluate patients’ liver and kidney function: Alanine Amiotransferase (ALT), Aspartate Aminotransferase (AST), Total Bilirubin (TBil), Serum Creatinine(SCr), Urea nitrogen (BUN), Glomerular Filtration Rate(GFR), 24 h before and 24 h after surgery, respectively.

Considering that the Bispectral Index (BIS) was originally developed for propofol, and studies have shown that the correlation between depth of sedation and the BIS index was weaker for the benzodiazepine agonist midazolam [9–12], we decided to use Modified Observer’s Assessment of Alertness/Sedation (MOAA/S) scale alone to evaluate the depth of sedation [13].

### Spinal anesthesia

We used combined spinal-epidural anesthesia for analgesia. Spinal puncture was performed with AS-E/SII needles at L3/4 in lateral decubitus position. After confirmation of clear and free-flow CSF, 3 ml of 0.5% ropivacaine was administered intrathecally over 10–15 s. After drug injection was completed, an epidural catheter was routinely placed in the epidural space to facilitate supplemental analgesia intraoperatively. The sensory block level of spinal anesthesia was evaluated every 2 min by pin-prick tests. After the peak sensory block level was determined, we used a modified Bromage Scale [14] (Table 1) to assess the degree of motor block. The lithotomy position was done for surgical preparation when the degree of anesthesia met surgery demand.

**Table 1:** Modified Bromage Scale [14]

Score	Criteria
1	Complete block(unable to move knee or feet)
2	Almost complete block(able to move feet only)
3	Partial block(able to move knee only)
4	Detectable weakness of hip flexion while supine(full flexion of knee)
5	No detectable weakness of hip flexion while supine

### Intervention and observed indicators

We used a modified Dixon’ up-and-down method to determine the and ED_50_ and ED_95_ of remimazolam tosilate to obtain a moderate sedation level of an MOAA/S 3/2 [15]. Based on previous literature and our pilot experiments, the initial remimazolam tosilate dose was 0.1 mg/kg [16]. After completing the an intermittent bolus of remimazolam infusion, a second anesthesiologist who didn’t know the dose of the trail drug evaluated the MOAA/S scales and vital signs every 1-min interval for the 10 min. If the patient responded only after his name was spoken loudly and/or repeatedly or responded only after mild prodding or shaking (MOAA/S scales 3/2) at any time point of assessment, we defined it as a successful sedation (Table 2). If targeted sedation (1 < MOAA/S scale < 4) was not obtained, we defined it as a failed sedation. According to the responses, the subsequent dose of RT was increased or decreased by 0.01 mg/kg for the next patient in a stepwise manner. Recruitment continued until ten independent pairs (from successful sedation to failed sedation) would give a reliable estimation of the moderate sedation dose of remimazolam tosilate.

**Table 2:** Modified Observer’s Assessment of Alertness/Sedation (MOAA/S) scale [15]

Scale	MOAA/S Scale
0	Does not respond to painful trapezius squeeze
1	Responds only after painful trapezius squeeze
2	Responds only after mild prodding or shaking
3	Responds only after name is called loudly and/or repeatedly
4	Lethargic response to name spoken in normal tone
5 (alert)	Responds readily to name spoken in normal tone

Mean arterial pressure (MAP), RR, HR, and SpO_2_ were recorded every minute. When a patient’s HR was less than 50 beats per minute (bradycardia) or whose MAP was lower than 20% of the baseline value (hypotension), he would be injected with 6 mg of ephedrine or 1 mg of atropine intravenously. If a patient's SpO_2_ was less than 90%, emergency ventilation would be performed including oxygen delivery via a face mask.

### Statistical analysis

Statistical analysis was performed using SPSS Statistics 25™ (SPSS Inc., Chicago, IL, U.S.A.). Investigations were carried out by Dixon’s up-and-down method. Up-and-down data were analyzed using the probit analysis to interpolate ED_50_ (95% C.I.) and ED_95_ (95% C.I.). Based on the probabilities calculated from the probit model, the fitted equations were derived and the dose effect curves were plotted.Values were expressed as the mean ± standard deviation (SD), mean (95% C.I.), or as numbers. The sample size was based on Dixon’s method, which requires at least six pairs of failure-success to calculate half maximal effective concentration (EC_50_) [17]. Patients were recruited until ten pairs of consecutive up and down (success and failure) adjustment of the remimazolam tosilate dose was achieved. Paired t-test was applied to count the changes of liver and kidney function before and after drug application. Statistical significance was defined by a *P* value < 0.05.

## Results

This study enrolled 30 patients. The demographic characteristics of the patients are shown in Table 3. During the design phase, we inclined to choose patients with similar height, weight, and BMI.

**Table 3:** Demographic characteristics of patients

characteristics	*n* = 30
Age (year)	74.80 ± 5.96
Height (cm)	165.60 ± 7.44
Weight (kg)	60.47 ± 11.08
BMI (kg/m^2^)	21.87 ± 2.33
ASA rating (I/II) (%)	9 (30%)/ 21 (70%)

**Fig. 1:**
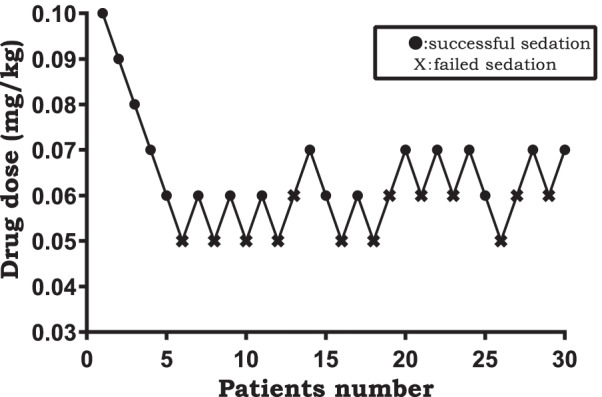
Dixion’s up-and-down method; success (mark●), failure (mark X)

**Fig. 2:**
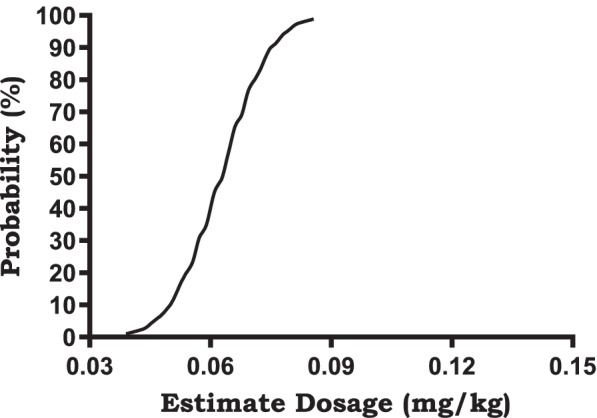
The dose–effect curve, Probit (*p*) = 19.485 + 16.148X (The covariate X, which represents the estimate dosage, is converted using a logarithm with base 10)

The sequences of successful and failed sedation are presented in Fig. 1. The estimated ED_50_ of remimazolam tosilate was 0.063 mg/kg (95% C.I. 0.045–0.073 mg/kg). The estimated ED_95_ was 0.079 mg/kg (95% C.I. 0.070–0.137 mg/kg). The dose–effect curve is shown in Fig. 2 with assigned estimate dosages (x-axis) and their respective probability (y-axis).

**Fig. 3:**
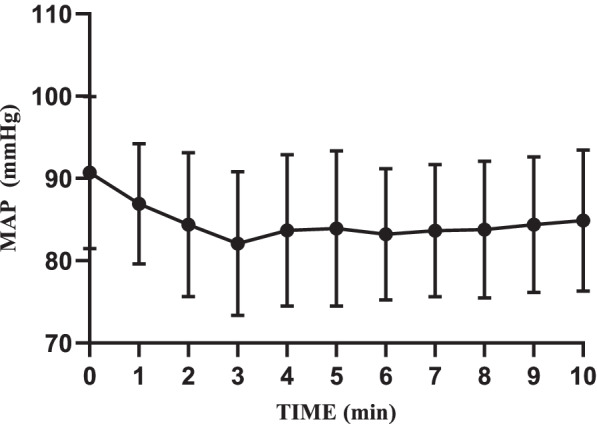
Intraoperative MAP changes

**Fig. 4:**
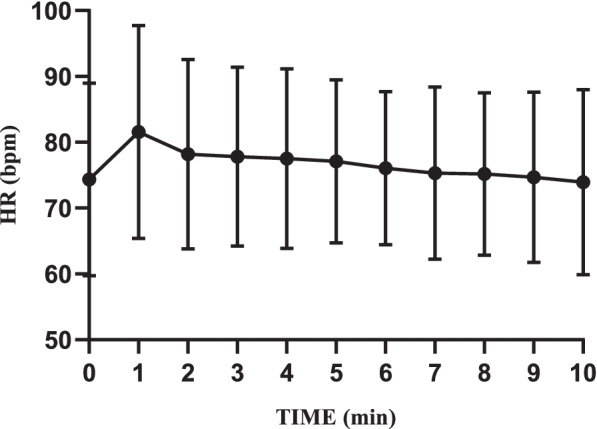
Intraoperative HR changes

**Fig. 5:**
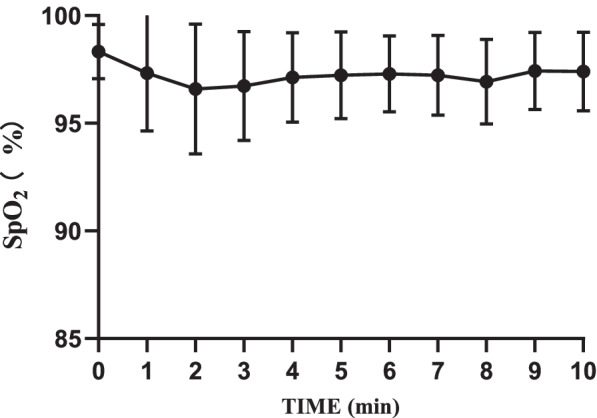
Intraoperative SpO_2_ changes

No patient experienced SpO_2_ < 90% during the trial. Hemodynamic parameters were stable (at 20% of baseline levels). No adverse events such as hypotension, bradycardia, respiratory depression, low blood oxygen saturation, injection pain, nausea and vomiting were found (Figs. 3, 4 and 5).

## Discussion

In this single-arm, single-centre clinical study, we obtained ED50 and ED95 of remimazolam tosilate in elderly patients undergoing TURP under spinal anesthesia and evaluated its efficacy and safety. Among the US and Chinese population, the number of people younger than 65 years of age is increasing by 1% per year, that aged 65–79 years is increasing by more than 2% annually and that aged 80 or older is increasing by 3% every year. According to demographic statistics, although the fastest growing segment in the world is people aged 80 years and older, its proportion remains relatively small. Therefore, we limited the age criteria between 65 and 80 in this trial. Elderly males who underwent TURP were included [1, 18, 19].

Different from other common sedative drugs, including propofol, remimazolam tosilate is a short-acting new benzodiazepine for IV sedation in limited duration procedures, such as upper gastrointestinal endoscopy, colonoscopy, closed reductions of long-bone fractures, and reductions of dislocations [20, 21]. Since we were aware that benzodiazepines exert little effect on the circulation and the respiratory system in elderly patients, we had confidence in the safety of anesthesia. Flumazenil is a specific antagonist of benzodiazepines and RT [20]. We were not concerned about adverse events led by excessive sedation during the design phase. RT is an ultra-short-acting agent for induction and maintenance of anesthesia, and for procedural sedation [8]. During our pilot experiments, almost all patients achieved an MOAA/S score of 4 at 7-min post administration. Thus, we ended the experiments 10 min after administering the agent. As expected, no patient required flumazenil to antagonize remimazolam tosilate, proving RT’s safety and metabolic stability.

We monitored BIS index during sedation in the pilot experiment phase. However, it was poorly correlated with the MOAA/S scale which showed a stronger association with the depth of sedation throughout the treatment with remimazolam tosilate. A study by the American Society of Anesthesiologists found marked heterogeneity of BIS scores, making it difficult to predict the depth of sedation. Originally developed for propofol, its relation with depth of sedation may not be independent of anesthetic agent [9–12]. Considering that our study was a single-arm trial of an ultra-short-acting benzodiazepine with rapid onset and short duration of action, we assessed patients’ depth of sedation in a more direct fashion rather than using BIS index. Spinal anesthesia may affect patient consciousness [22]. The effect was correlated with level of block. This translated into a positive association between anesthesia plane and degree of sedation [23]. Based on these results, we strictly followed the modified Bromage Scale to assess analgesia and strived to ensure sample homogeneity.

One characteristic of the modified Dixon’s up-and-down method is determining the dose for the next patient according to that of the previous patient, resulting in two different doses. In this way, reliable conclusions could be drawn with a smaller sample size, which not only saves manpower and time, but also avoids applying immature methods in a large group of patients. Easy-to-adjust dosages and fast-to-obtain results are prerequisites of this sequential method. Regarding the sample size calculation, we strictly adhered to the principle of at least 6 pairs in order to calculate the ED95 and ED50 of remimazolam tosilate more rigorously, based on the advantage of a Dixon’s up-and-down method with significant sample size savings. Studies by the team of Professors Stylianou M and Dixon WJ on Up-and-down method and Isotonic Regression have shown that a linearly interpolated isotonic regression estimate is shown to be simple to derive and to perform as well as or better than the other target dose estimators in terms of mean square error and average number of subjects needed for convergence in most scenarios studied. Most importantly, Dixon’s up-and-down method are based on the response of pre- and post-trial subjects to different trial drugs to determine the subsequent trial drug dose, and based on this characteristic we were also unable to calculate a specific sample size [15, 17]. In this study, the dose of RT was reasonably selected according to relevant literature and previous studies conducted in the Second Affiliated Hospital of Hainan Medical University. Besides, probit analysis is widely used to calculate the ED_50_ and the ED_95_ of drugs.

In this single-arm, single-centre clinical trial, we obtained the ED_50_ and the ED_95_ for single-dose remimazolam tosilate in elderly patients undergoing TURP with spinal anesthesia for moderate sedation, which were 0.063 (95% C.I. 0.045–0.073) mg/kg and 0.079 (95% C.I. 0.07–0.137) mg/kg, respectively. Used in minor surgeries with a short duration, one disadvantage of benzodiazepines that cannot be ignored is that they have no analgesic effect. Therefore, we combined analgesia and anesthesia for our trial. We compared our study with another trial conducted by Peking University First Hospital (Sheng and Liang, 2020). They applied remimazolam in healthy Chinese volunteers [16]. Despite our differences in the method of anesthesia and targeted population, the two studies shared similar results in the efficacy and safety of remimazolam tosilate. With spinal anesthesia, we managed to free all male patients undergoing TURP from pain.

Manufacturer suggests that sedation with remimazolam tosilate can be achieved with 5 mg loading dosage for all patients regardless of their weight. According to the experimental results provided by the manufacturer, RT has linear pharmacokinetics which is independent of body weight [24]. However, as per the pre-designed dosage scheme, doses for a significant proportion of our patients did not reach the above value after calculation. Therefore, when administered with the recommended dosage under a completely painless state, patients are prone to excessive sedation. The study mentioned above explored safety, pharmacokinetic and pharmacodynamic properties of single ascending dose and continuous infusion of remimazolam besylate in healthy Chinese volunteers (Sheng and Liang, 2020) [16]. Results showed that the sedation was initially observed at the dose of 0.05 mg/kg. The agent exerted its peak effect at a ≥ 0.075 mg/kg dosage within 1–2 min after injection with a deeper sedation and a more rapid induction. Therefore, to avoid excessive sedation, the minimum optimal dose of remimazolam tosilate should be determined. In clinical practices, we should consider patients’ age, physical condition, and pain and stress caused by surgical operations. Since no intravenous analgesic that maintains spontaneous respiration is as effective as general anesthesia assisted with tracheal intubation, we hope that our trial can provide an alternative plan for anesthesiologists.

According to related studies at home and abroad on the same agent, as an anesthetic sedative drug, remimazolam tosilate shows a satisfactory safety profile in endoscopic procedures [6, 8, 11, 12]. We tried to validate its safety by comparing levels of main vital signs such as MAP, SpO_2_, and HR (Figs. 3, 4 and 5). No obvious changes were observed in the curves. Throughout the trial, we found no serious adverse events or adverse reactions which required intervention. It is worth mentioning that all patients breathed indoor air autonomously. No oxygenation devices were needed. Compared with T_0_, although SpO_2_ levels decreased to a certain extent after administration, especially within the first two minutes, the minimum value was still much higher than the clinical lower limit of normal. This proves that remimazolam tosilate has little inhibition on the respiratory system. All patients were able to achieve a MOAA/S score of 5 within 10 min after a single dose of remimazolam tosilate, so we did not use flumazenil for additional drug antagonism. However, flumazenil can be readily applied to patients as a safety measure. During the whole procedure, no injection site pain was observed, which was consistent with previous studies [6, 8, 25].

In terms of laboratory results of liver and kidney function, remimazolam tosilate continues to show a relatively promising clinical safety profile (Table 4). there was no statistically significant difference in the results of transaminases (*P* > 0.05). As mentioned in the manufacturer's instructions, we found an increase in bilirubin in the laboratory test results. Considering that the patient's postoperative bilirubin index had a mild increase (*P* < 0.05), but no patient exceeded the upper limit of the test value. Moreover, the patient was routinely given antibiotics and other medications both intraoperatively and postoperatively, so it is not entirely clear that remimazolam tosilate had a negative effect on the patient's liver function. However, assessing the performance of the laboratory tests for transaminases and bilirubin together with the previous tests related to the comparison of remimazolam tosilate with other sedative drugs, we can confirm that the effect of remimazolam tosilate on the liver is very mild [20, 21, 24, 25]. We analyzed the performance of the patients' kidney function and could find a statistically significant improvement in all the indicators related to kidney function (*P* < 0.05). However, we cannot ignore that the impact of TURP on patients' kidney function is tremendous, and it can largely alleviate the phenomenon of urinary obstruction in patients. Therefore, we cannot easily and completely affirm or deny the effect of remimazolam tosilate on patients’ renal function through our experimental design, but more in-depth studies are needed.

**Table 4:** Laboratory tests of the patient’s liver and kidney function

characteristics	24 h before surgery (*n* = 30)	24 h after surgery (*n* = 30)	*P* value
ALT (u/L)	17.67 ± 4.999	17.57 ± 5.900	*P* = 0.921
AST (u/L)	18.27 ± 5.092	19.03 ± 4.916	*P* = 0.549
TBil (μmol/L)	11.02 ± 6.403	12.87 ± 4.834	*P* = 0.024
sCr (μmol/L)	77.33 ± 27.493	71.03 ± 22.630	*P* = 0.018
BUN (mmol/L)	5.84 ± 1.858	4.79 ± 1.567	*P* < 0.001
GFR(ml/min)	82.80 ± 32.810	134.53 ± 65.589	*P* < 0.001

We used BIS monitoring on patients during the pre-experimental phase. During the study it was found that the bis values of the patients correlated poorly with the MOAA/S scores of the patients, which largely affected the course of our trial. We reviewed the relevant literature and many clinical trials of BIS monitoring for anesthetic drugs showed unsatisfactory results of bis monitoring in correlation with benzodiazepines [9–12, 26]. The issue of BIS and EEG for monitoring consciousness in patients undergoing anesthesia with remimazolam tosilate depends on further in-depth studies.

There are some limitations of this trial. This was a single-centre investigation with a relatively small sample size. Therefore, these results need to be validated in further research.

## Conclusions

A single-dose of remimazolam tosilate proves to be effective and safe for elderly patients undergoing TURP under spinal anesthesia. Its ED_50_ and ED_95_ were 0.063 (95% C.I. 0.045–0.073) mg/kg and 0.079 (95% C.I. 0.07–0.137) mg/kg, respectively. Meanwhile, adverse events, such as respiratory depression, hypotension and inject pain, are largely avoided. This study was a single-centre study. More studies are needed to validate the conclusion.

## Supplementary Information

**Additional file 1.** 

**Additional file 2.** 

**Additional file 3.** 

**Additional file 4.** 

## Data Availability

All data generated or analyzed during this study are included in this published article and its supplementary information files.

## Abbreviations

RT: Remimazolam tosilate
TURP: Transurethral resection of the prostate
MOAA/S: Modified Observer’s Assessment of Alertness/Sedation
ASA: American Society of Anesthesiologist
BMI: Body mass index
CSF: Cerebrospinal fluid
ECG: Electrocardiogram
NIBP: Noninvasive blood pressure
SBP: Systolic blood pressure
DBP: Diastolic blood pressure
SpO_2_: Blood oxygen saturation
RR: Respiratory rate
HR: Heart rate
BIS: Bispectral Index
